# Supplementary material for: Global analysis of the RNA–RNA interactome in Acinetobacter baumannii AB5075 uncovers a small regulatory RNA repressing the virulence-related outer membrane protein CarO
Source: Nucleic Acids Res. 2024 Aug 16;52(18):11283–300. doi: 10.1093/nar/gkae668 (PMC11472050; doi:10.1093/nar/gkae668)
Supplement: gkae668_Supplemental_Files [file gkae668_supplemental_files.zip › Supplementary Figures.pdf]

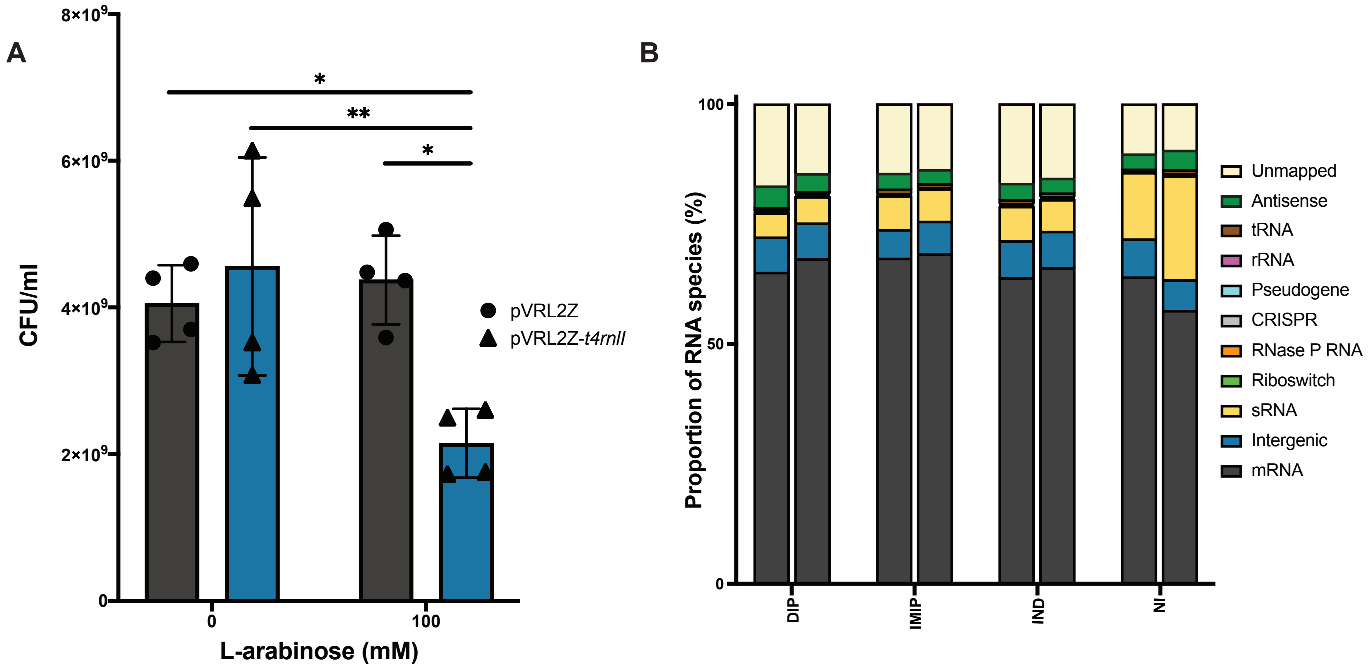

Supplementary Figure 1: Hi-GRIL-seq in *A. baumannii* AB5075. (A) Viability experiment to identify the concentration of L-arabinose that caused a reduction in cell density without causing excessive cell death. Colony forming units (CFU/ml) of strains carrying the pVRL2Z-*t4rnlI* plasmid and strains carrying the empty control vector (pVRL2Z) plasmid were measured following 1 h induction with 0 mM or 100 mM L-arabinose once the cultures reached early stationary phase (ESP, OD_{600} 2.0). Error bars represent the standard deviation from four independent biological replicates. Statistical comparisons were performed using Two-way ANOVA followed by Tukey's multiple comparisons test. Differences were considered statistically significant where * denotes P<0.05 and ** denotes P<0.01. (B) The percentage of RNA species identified by Hi-GRIL-seq in iron starvation (DIP), imipenem shock (IMIP), the induced sample (IND) and the uninduced negative control (NI) treatment conditions. Results from independent biological duplicates of each treatment condition is visualised as adjacent bars. The sum of the RNA species percentage is >100% due to the presence of sequencing reads derived from regions with overlapping annotations.

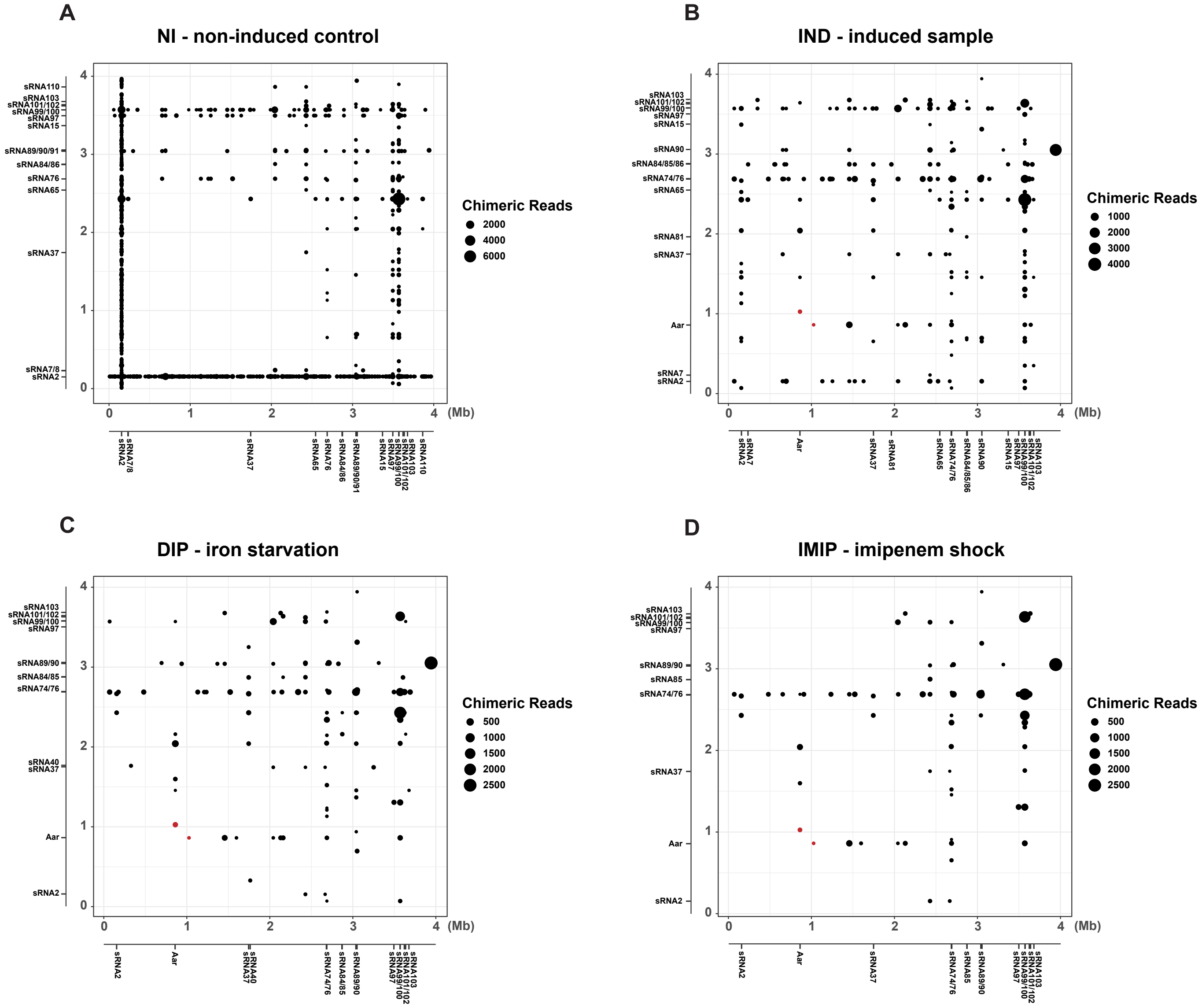

Supplementary Figure 2: Global identification of sRNA-RNA interactions identified by Hi-GRIL-seq in *A. baumannii* AB5075 in different conditions. The genomic locations of sRNA-containing chimeric reads $\geq$50 sequencing reads were mapped to the AB5075 chromosome in the (A) T4 RNA ligase uninduced (NI) control condition, (B) T4 RNA ligase induced (IND) condition, (C) iron starvation (DIP) condition and (D) imipenem shock (IMIP) condition. The x-axis represents the $5'$ end of each chimeric fragment while the y-axis represents the $3'$ end of the fragment. Each dot represents the exact genomic coordinate of these fragments. The size of the dot is proportional to the number of sequencing reads observed. The location of all sRNA candidates contained within RNA-RNA chimeras are shown. The locations of the Aar-*carO* mRNA chimeric sequencing reads are labelled in red.

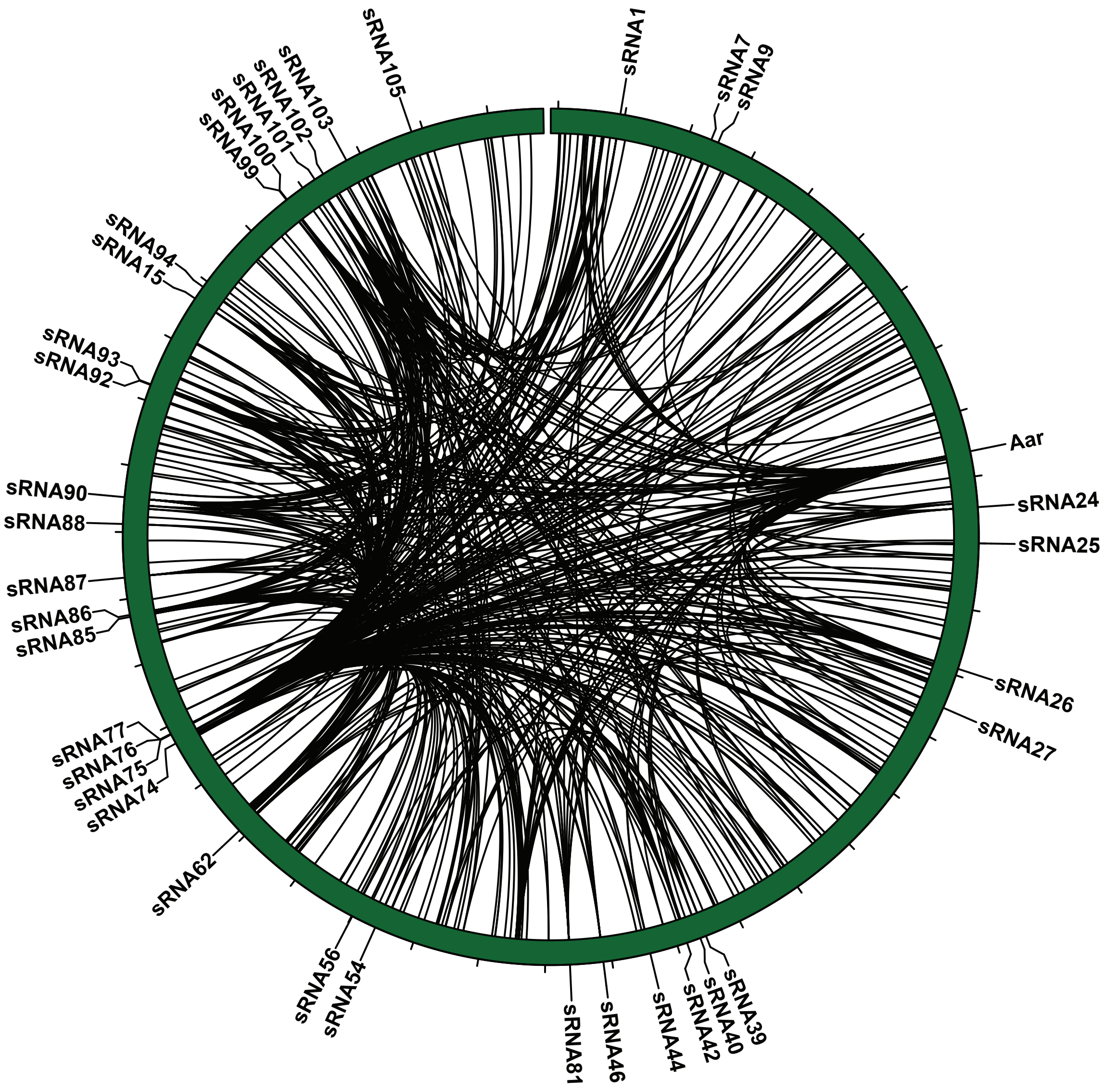

Supplementary Figure 3: Chromosomal sRNA-RNA interactions that are overrepresented in the T4 RNA ligase induced conditions (IND, DIP and IMIP). Analysis Hi-GRIL-seq revealed that 36 *A. baumannii* AB5075 sRNA candidates were ligated to RNA molecules in other regions of the chromosome. This resulted in the identification of 632 potential chromosomal sRNA-RNA interactions that matched the cut-off scores applied. These interactions are visualised for each sRNA candidate across the AB5075 chromosome.

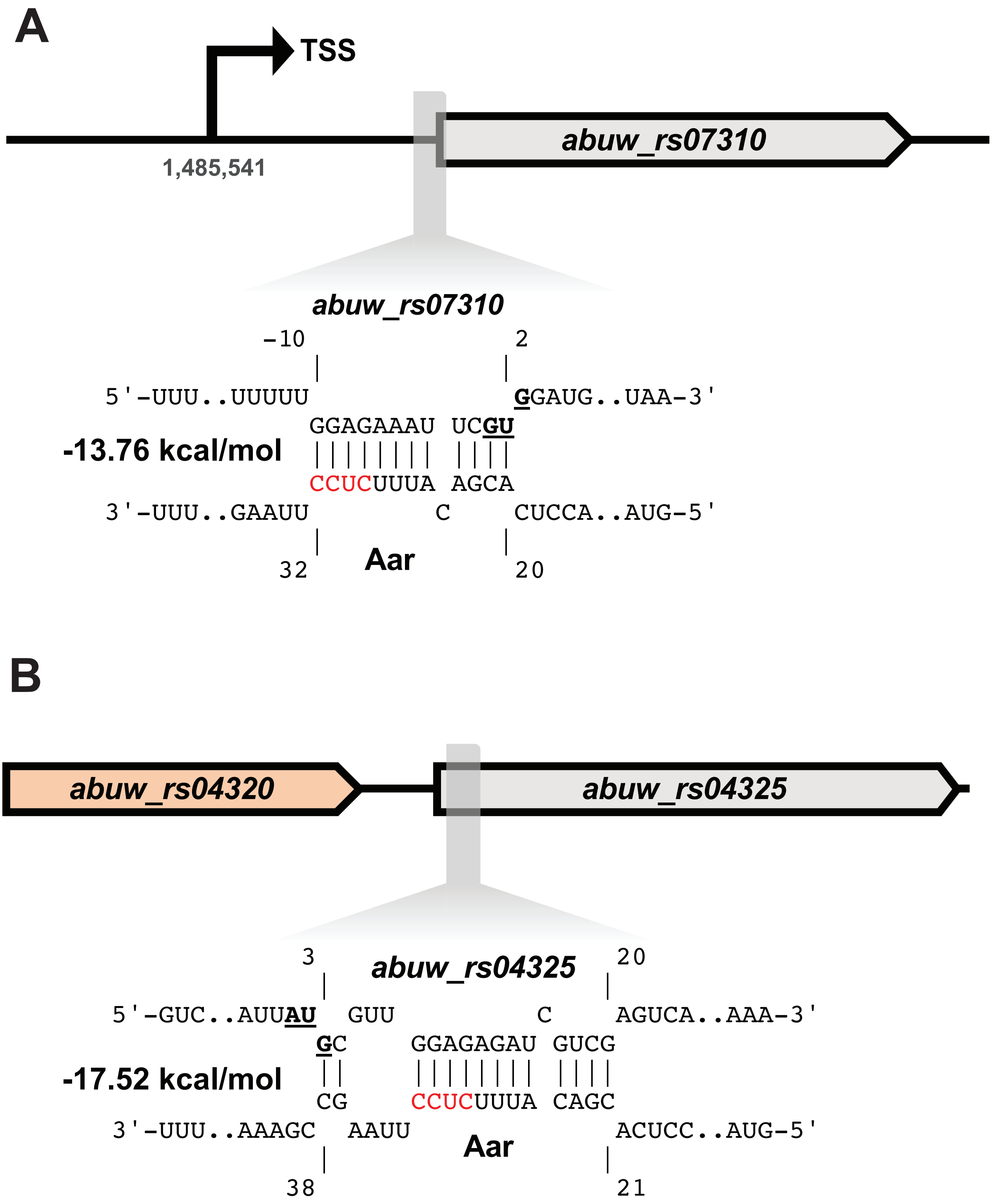

Supplementary Figure 4: Prediction of Aar-mRNA interactions identified by Hi-GRIL-seq identifies a possible Aar seed region. The sRNA-mRNA interaction prediction tool IntaRNA was used to identify likely interactions between Aar and Hi-GRIL-seq derived mRNA targets. The position of the IntaRNA predicted interaction for each target is shown on the chromosome for (A) *ABUW_RS07310* and (B) *ABUW_RS04325*. The position of the TSSs is shown as curved arrow. The location of the predicted interactions is shown relative to the start codon of mRNA targets. The start codon is in bold and underlined. The predicted Aar "seed" region (5′-CUCC) is highlighted in red.

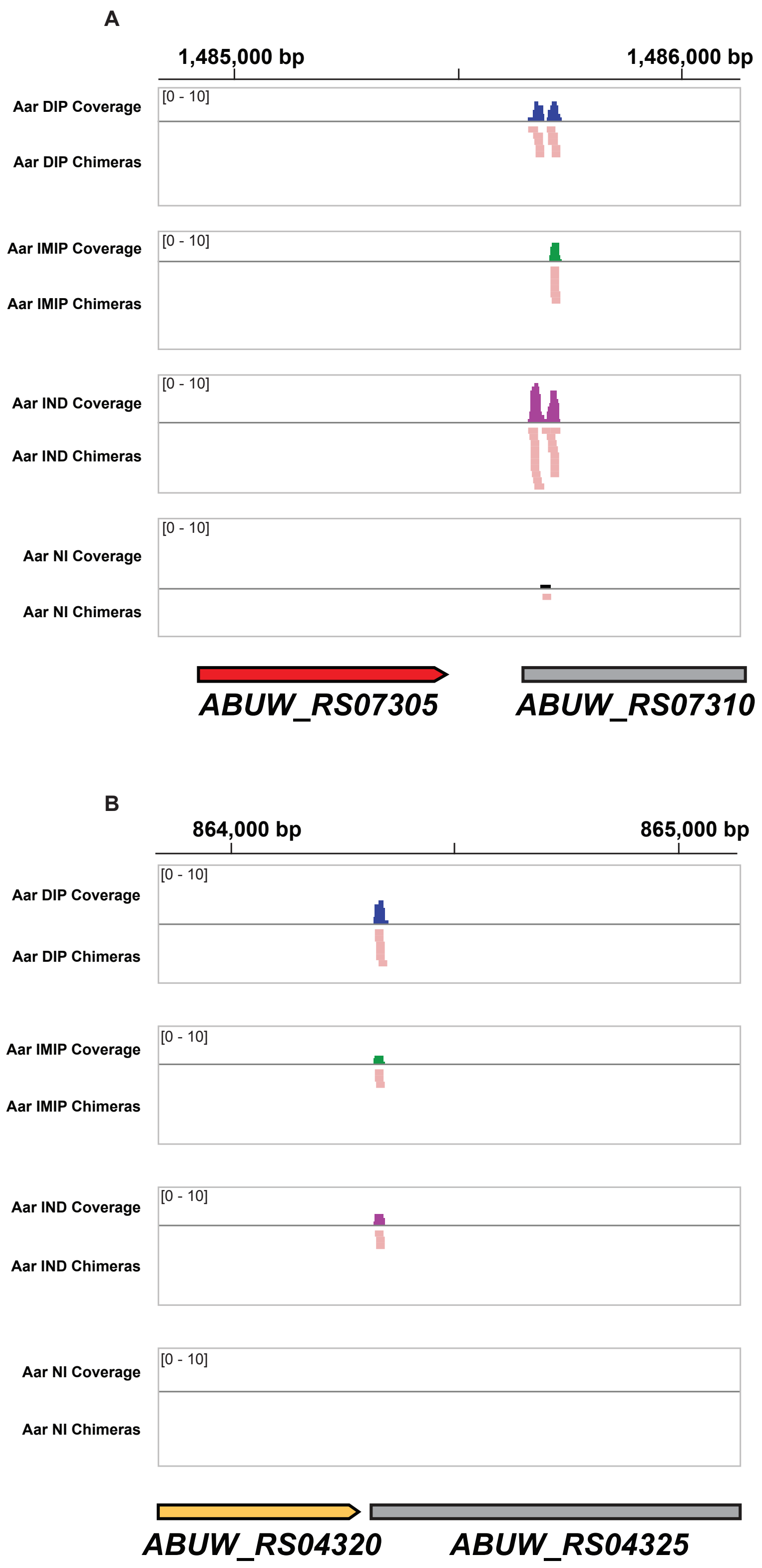

Supplementary Figure 5: Location of Aar-*ABUW_RS07310*/*ABUW_RS04325* chimeric reads mapping to the AB5075 chromosome. The location of the (A) *ABUW_RS07310* and (B) *ABUW_RS04325* portions of Aar-containing chimeric fragments were mapped to the AB5075 for each Hi-GRIL-seq condition. To facilitate visualisation of the chimeric reads, the reads from the two biological replicates of each condition were combined. A coverage profile of the number of chimeric reads is shown on the upper part of each track. Individual chimeric reads are shown in the lower part of each track.

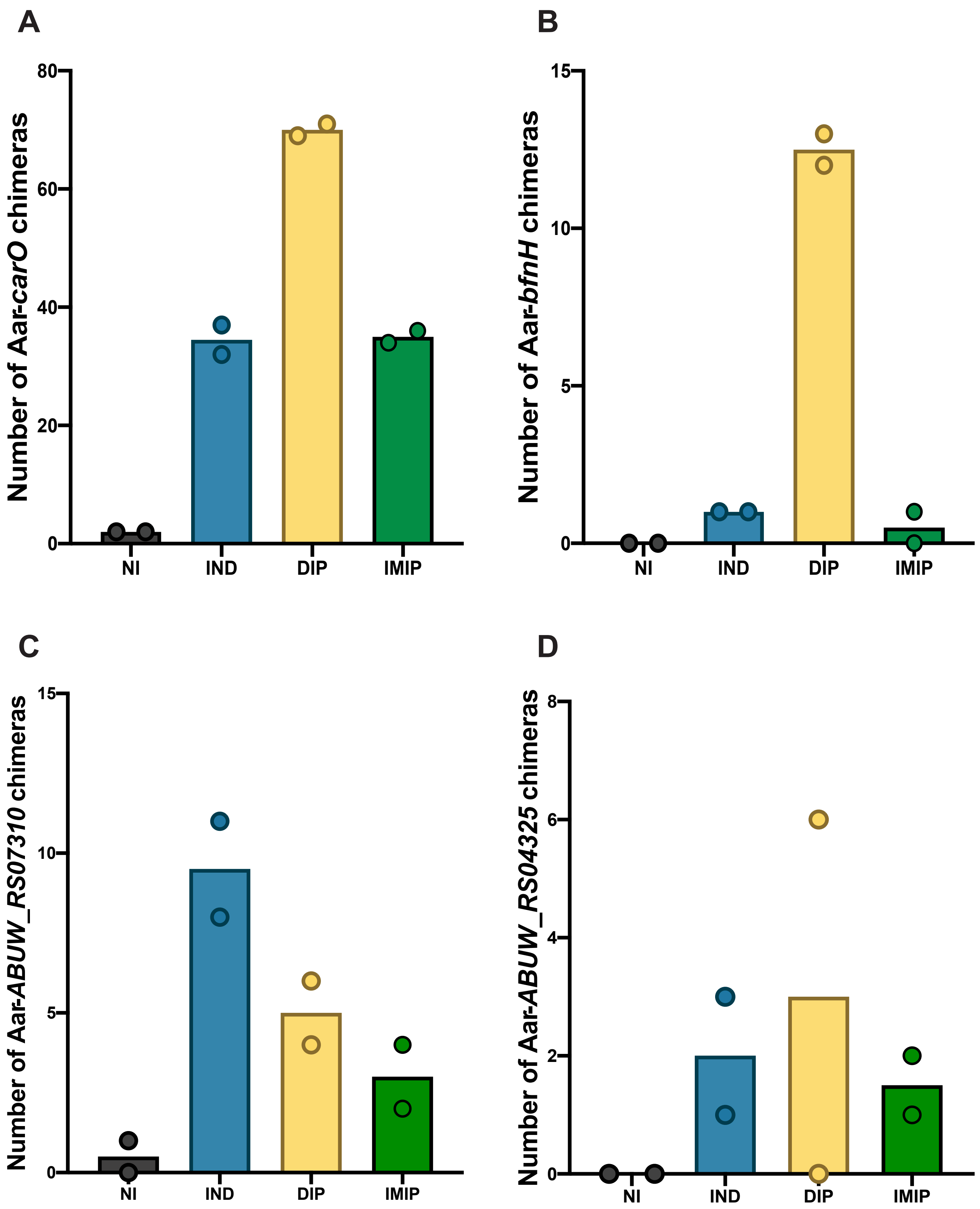

Supplementary Figure 6: Characterising the Hi-GRIL-seq conditions in which Aar-mRNA chimeric reads are most abundant. Depicted are the conditions in which (A) Aar-*carO*, (B) Aar-*bfnH*, (C) Aar-*ABUW_RS07310* and (D) Aar-*ABUW_RS04325* chimeric molecules were most abundant in Hi-GRIL-seq. We show the number of chimeras identified in the non-induced (NI) control and the induced (IND), iron starvation (DIP) and imipenem shock (IMIP) conditions. We show the mean of the two biological conditions while the individual points represent the number of chimeras identified in each biological replicate.

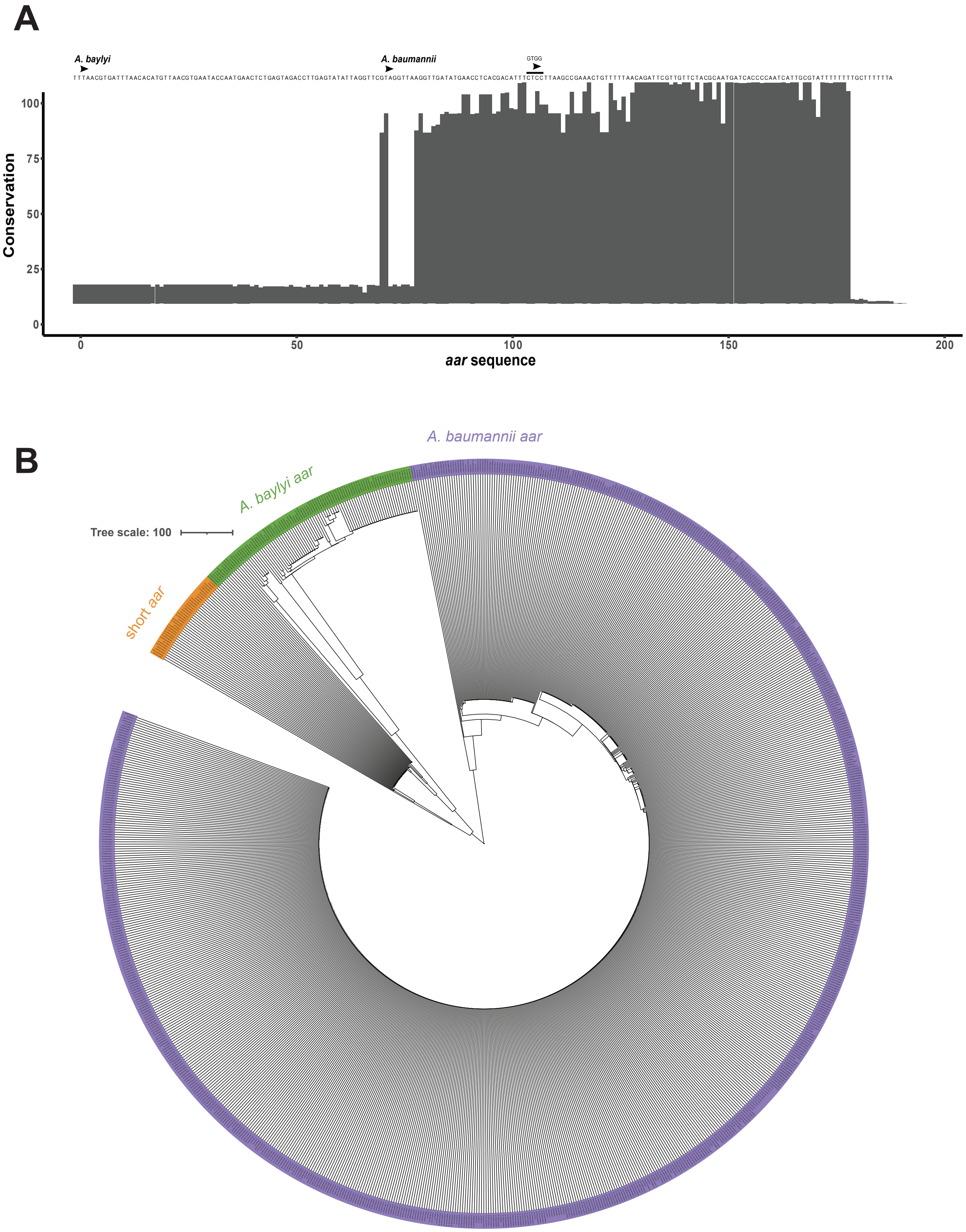

Supplementary Figure 7: *aar* forms separate clusters in *A. baumannii* and *A. baylyi*. (A) Sequence conservation analysis of the *aar* locus in *A. baumannii* and *A. baylyi* strains showing the extent of sequence divergence in *aar*. The location of the predicted *A. baumannii* and *A. baylyi* TSSs and the seed region are shown as arrows. The mutated form of the seed region is indicated. (B) Phylogenetic visualisation of *aar* conservation within *A. baumannii* and *A. baylyi*. These organisms form separate groups where a subgroup of *A. baylyi* contains a shorter *aar* sequence.

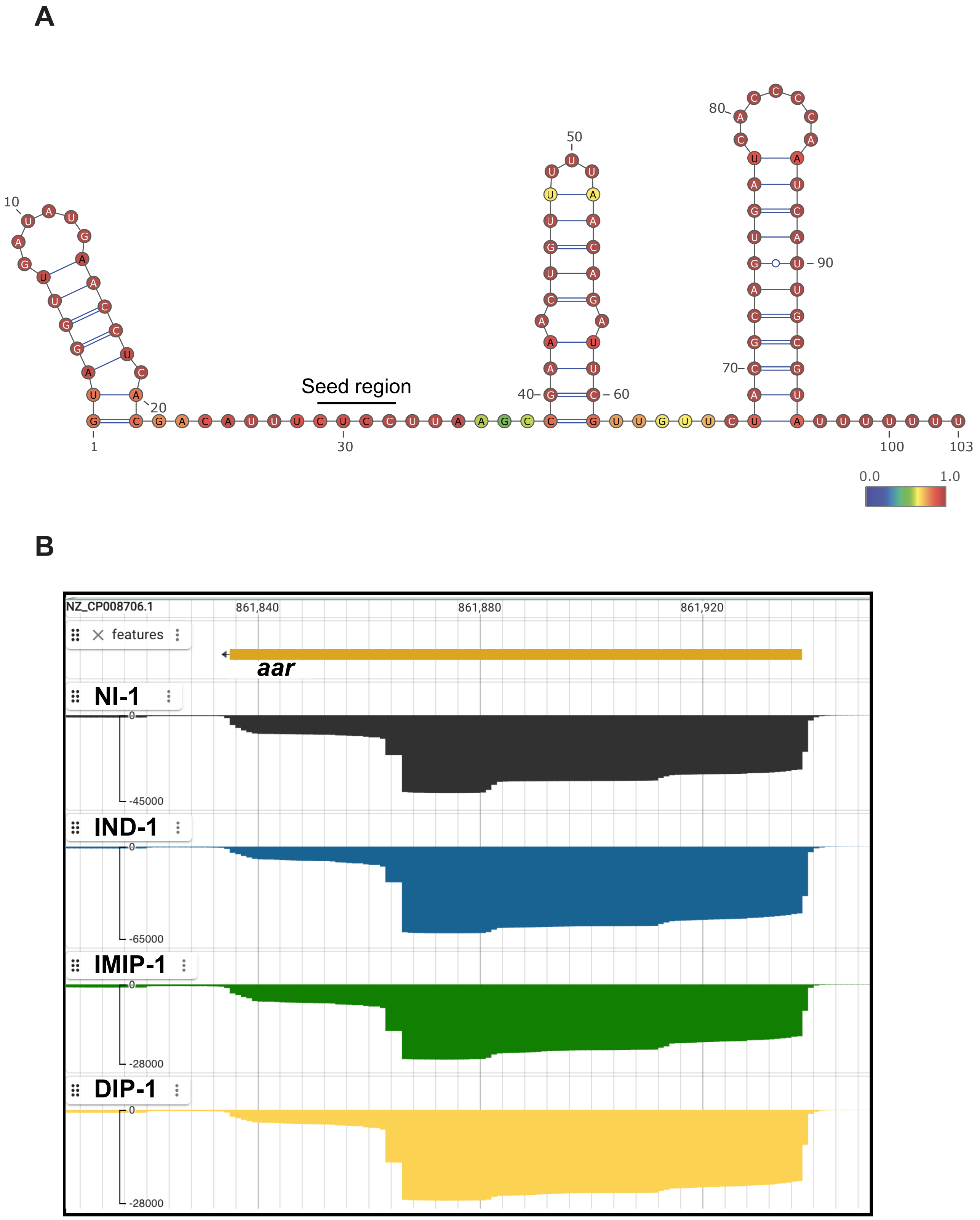

Supplementary Figure 8: Predicting the secondary structure of Aar. (A) The secondary structure of full-length Aar was predicted using RNAfold and was visualised using VARNA showing the predicted minimum free energy (MFE) structure (Darty *et al.*, 2009; Lorenz *et al.*, 2011). Individual nucleotides are coloured based on their base-pairing probabilities. The Aar seed region is highlighted. (B) The read coverage of the *aar* locus in the Hi-GRIL-seq mapped reads is visualised in JBrowse2 (Diesh *et al.*, 2023). The sequencing reads are shown for one biological replicate of each Hi-GRIL-seq condition tested (NI = non-induced control, IND = induced sample, DIP = iron starvation and IMIP = imipenem shock). The mapped sequence read data can be viewed at: http://bioinf.gen.tcd.ie/jbrowse2/Hi-GRIL-seq.

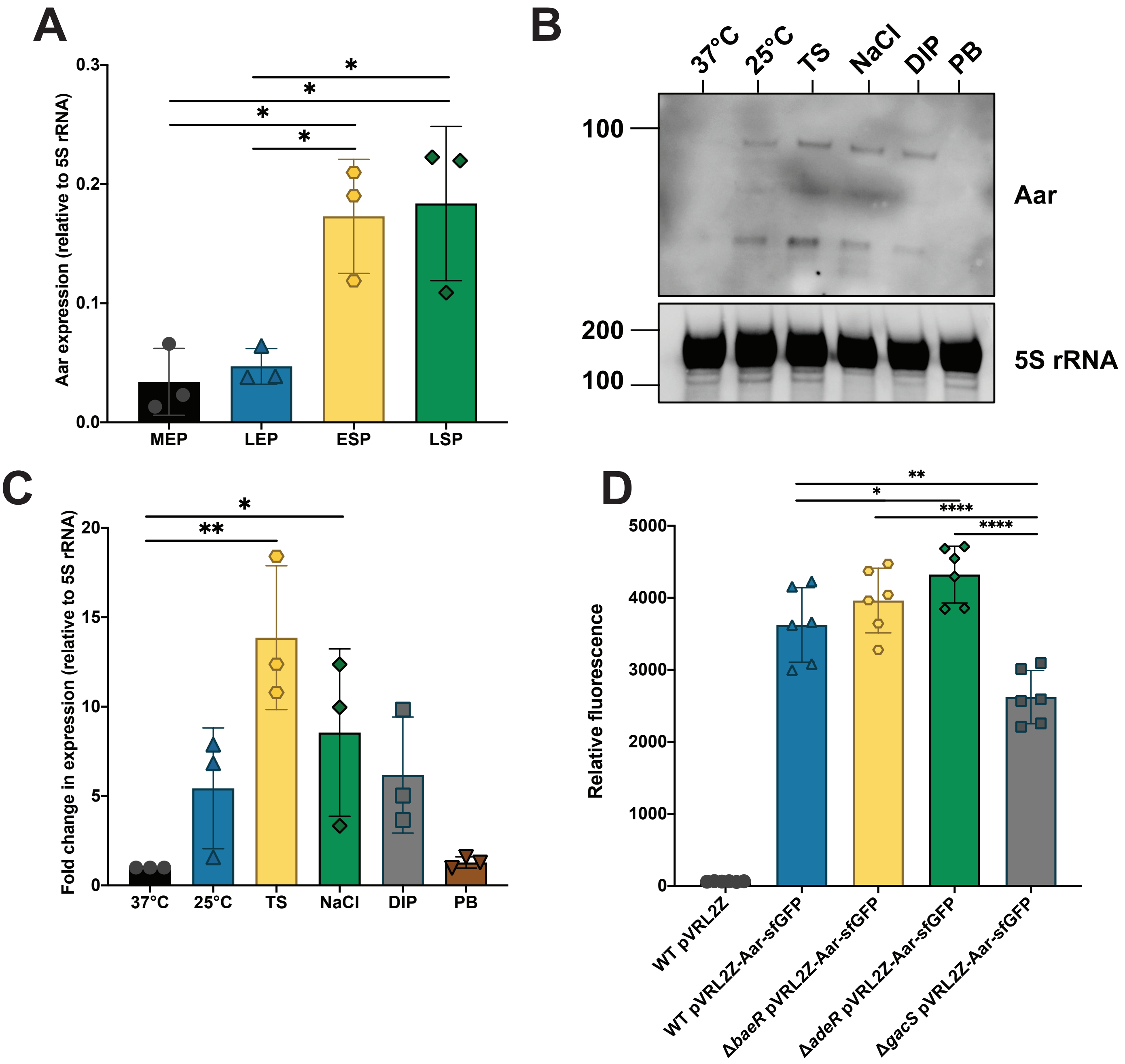

Supplementary Figure 9: Aar expression accumulates across growth and is induced by different environmental conditions. (A) Quantification of Aar expression levels in *A. baumannii* across growth phases including; MEP, LEP, ESP and LSP, as determined by northern blotting. (B) The expression of Aar in *A. baumannii* in several different conditions was compared by northern blotting. *A. baumannii* was grown to LEP at either 37°C or 25°C. RNA was isolated from cells grown at 37°C that were shocked for 15 min with 0.3 M NaCl (NaCl), 200 µM 2,2-dipyridyl (DIP) or 1 µg/ml polymyxin B (PB) and from a mock treatment control grown at 37°C (37°C). RNA was also isolated from cells following a 15 min temperature shift (25°C to 37°C, TS) and from a mock treatment control grown at 25°C (25°C). Five µg of total RNA was used for northern blotting. Expression of Aar and the loading control, 5S rRNA, were detected using their respective DIG-labelled riboprobes in northern blotting experiments. This was performed three times independently, and one representative blot is shown. (C) Quantification of Aar expression levels in different shock conditions as determined by northern blotting. Aar expression levels were adjusted to the loading control (5S rRNA). The fold change in Aar expression was then compared to the control sample (37°C, set to 1). (D) An Aar transcriptional reporter in *A. baumannii* AB5075 reveals that Aar is regulated by AdeR and GacS. The relative fluorescence of the *aar* promoter transcriptionally fused to sfGFP (pVRL2Z-Aar-sfGFP) was measured in wildtype (WT) and deletion strains (Δ*baeR*, Δ*adeR* and Δ*gacS*) of *A. baumannii* at LSP. A wildtype strain carrying pVRL2Z was included to measure autofluorescence of *A. baumannii*. Statistical tests were performed using One-way ANOVA followed by Tukey's test (A and D) or Dunnett's test (C). Error bars represent the standard deviation from independent biological replicates. Differences were considered statistically significant where * denotes $P<0.05$, ** denotes $P<0.01$, *** denotes $P<0.001$ and **** denotes $P<0.0001$.

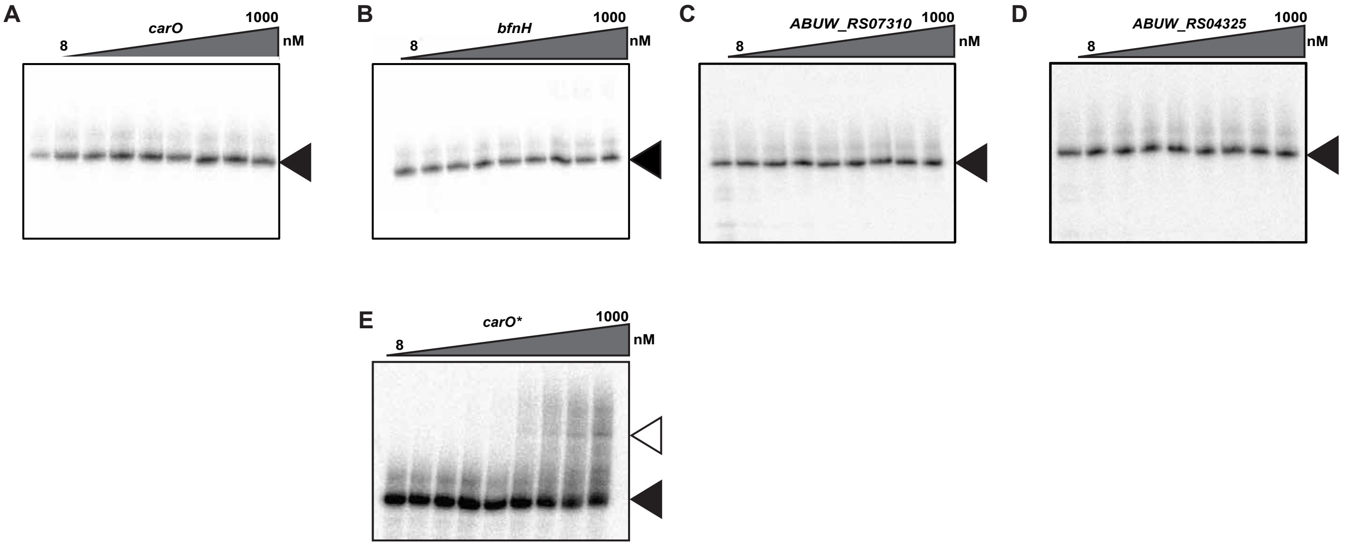

Supplementary Figure 10: Identification of Aar-mRNA interactions *in vitro* using EMSAs. The ability of Aar* to interact with (A) *carO*, (B) *bfnH*, (C) *ABUW_RS07310*, (D) *ABUW_RS04325* and (E) *carO** was assessed using EMSAs. This was accomplished by incubating radiolabelled Aar* in the absence and in the presence of increasing concentrations (from 8 nM to 1000 nM) of putative mRNA targets.

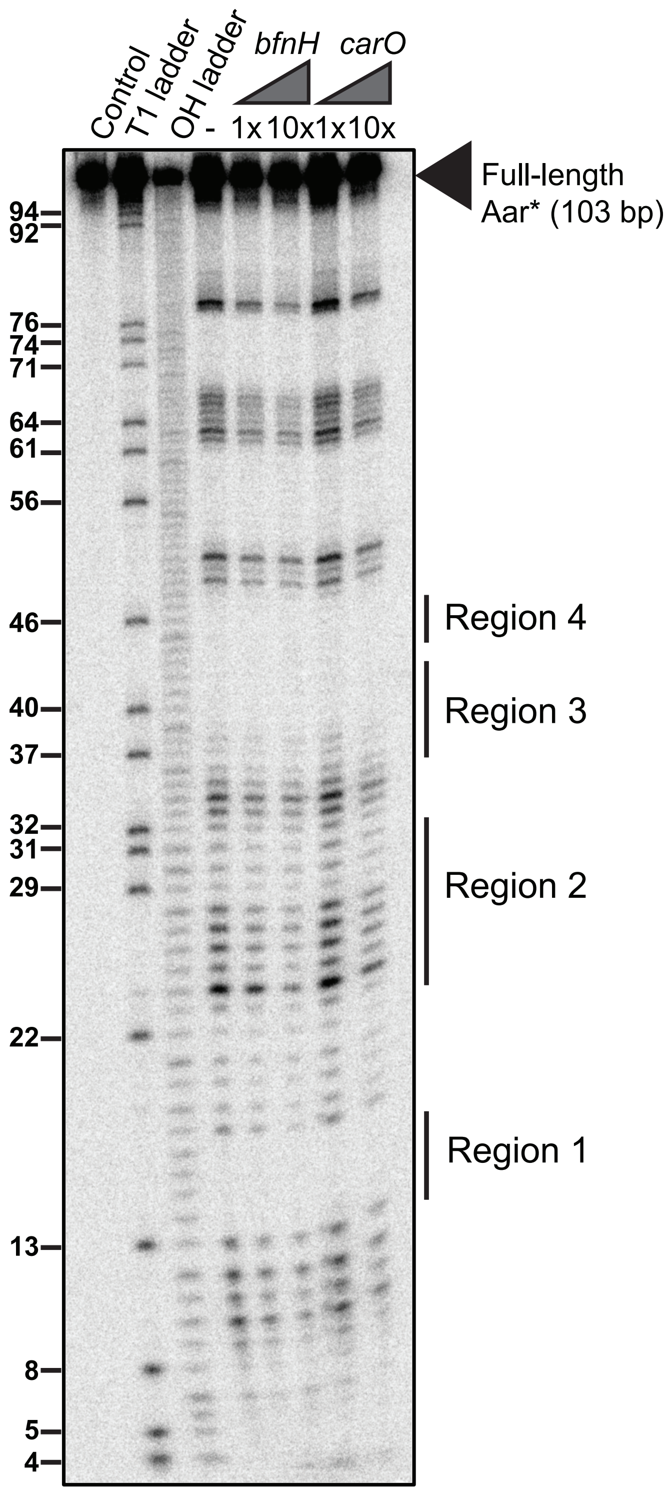

Supplementary Figure 11: Disruption of the Aar seed region inhibits Aar-mediated interactions *in vitro*. The effect of mutating the Aar seed region (Aar*) on base-pairing with *bfnH* and *carO* at the nucleotide level was examined using in-line probing. The regions of nucleotides that were protected in wild-type Aar in the presence of these mRNA interaction partners is highlighted (Regions 1-4). Two ladders were included; a T1 ladder, where the sRNA was cleaved at guanine nucleotides, and a OH ladder, which cleaved the sRNA at the nucleotide level. The Aar seed region nucleotide substitution is visible on the T1 ladder (nucleotides 29, 31 and 32). An uncleaved Aar* control was also included.

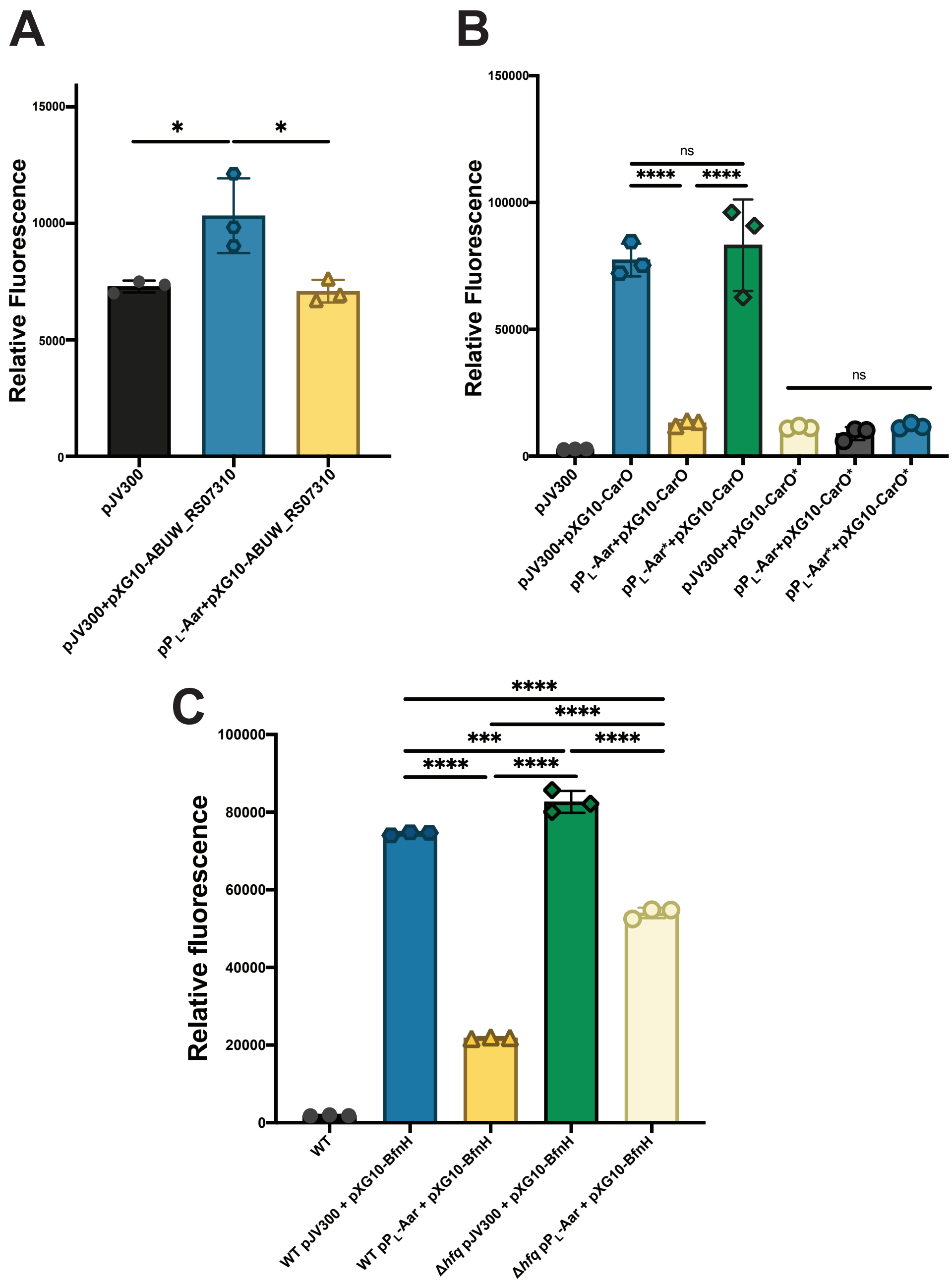

Supplementary Figure 12: Aar suppresses translation of ABUW_RS07310, sequesters the *carO* Shine-Dalgarno sequence and relies on Hfq in heterologous two-plasmid reporter systems. (A) The involvement of Aar in modulating expression of ABUW_RS07310 was assessed using a two-plasmid system in *E. coli*. The relative fluorescence intensity of strains carrying the pXG10-ABUW_RS07310 plasmid with either the control plasmid (pJV300) or an Aar overexpression vector (pP$_L$-Aar) was measured. A strain only carrying pJV300 was also included to measure autofluorescence of *E. coli*. Error bars represent the standard deviation from three independent biological replicates (n=3). (B) Disruption of *carO* nucleotides complementary (CarO*) to the Aar seed region disrupts fluorescence. The relative fluorescence intensity of strains carrying the pXG10-CarO* plasmid with either the control plasmid (pJV300), an Aar overexpression vector (pP$_L$-Aar) or an Aar* overexpression vector (pP$_L$-Aar*) was measured. The results from Figure 5A are also shown as comparators. Error bars represent the standard deviation from three independent biological replicates (n=3). (C) Hfq contributes to Aar-*bfnH* base-pairing in wildtype and Hfq-deletion (Δ*hfq*) *S.* Typhimurium 4/74 strains. The relative fluorescence intensity of strains carrying the pXG10-BfnH plasmid with either the control plasmid (pJV300) or an Aar overexpression vector (pP$_L$-Aar) was measured. Wildtype 4/74 was also included to measure autofluorescence of 4/74. Error bars represent the standard deviation from three independent biological replicates.Statistical comparisons were performed using One-way ANOVA followed by Tukey's multiple comparisons test. Differences were considered statistically significant where ns denotes P>0.05, * denotes P<0.05, ** denotes P<0.01, *** denotes P<0.001 and **** denotes P<0.0001.

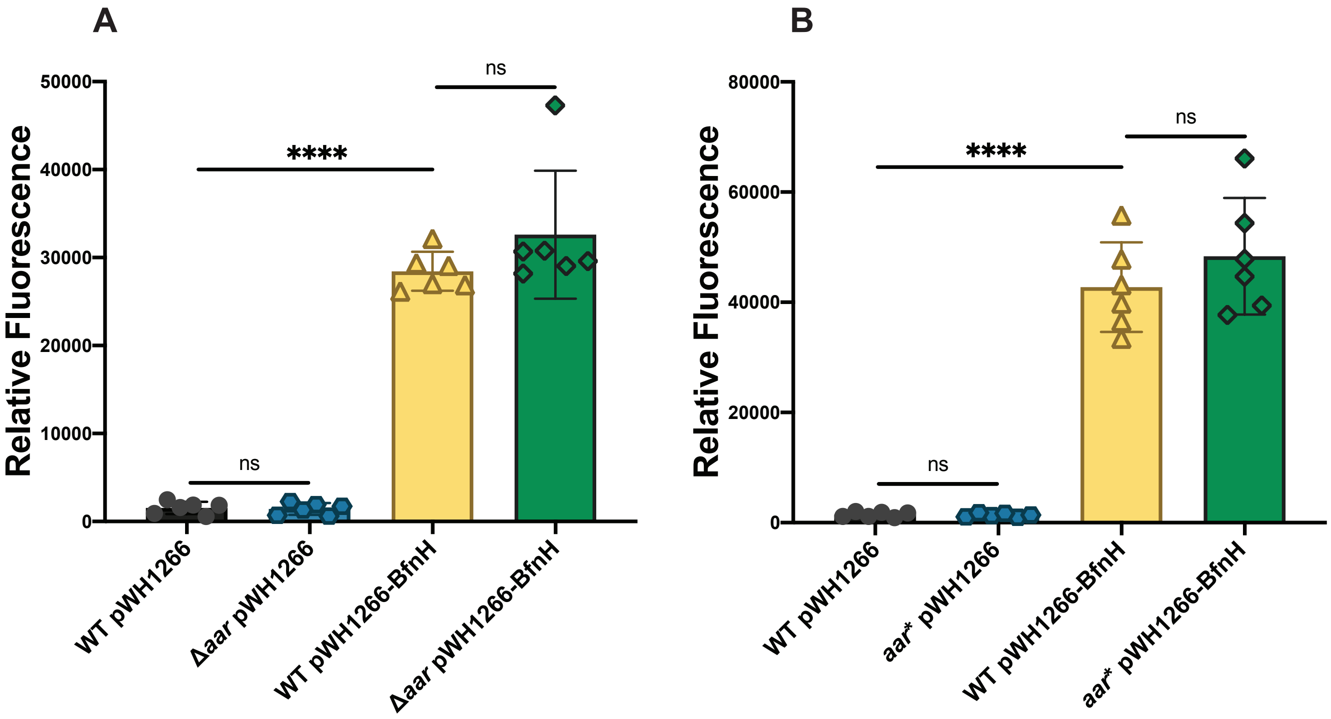

Supplementary Figure 13: Regulation of BfnH by Aar using a translational reporter in *A. baumannii* AB5075. The involvement of (A) Aar and (B) Aar* in modulating translation of BfnH-sfGFP was assessed in *A. baumannii* using a translational reporter. The relative fluorescence intensity of wild-type (WT), Aar-deletion (Δ*aar*) or Aar seed region mutant (*aar**) strains of *A. baumannii* AB5075 carrying the pWH1266-BfnH plasmid was compared at LSP. Control strains carrying the pWH1266 plasmid were also measured to quantify the autofluorescence of strains. Error bars represent the standard deviation from six independent biological replicates. Statistical comparisons were performed using One-way ANOVA followed by Tukey's multiple comparisons test. Differences were considered statistically significant where ns denotes P>0.05, * denotes P<0.05, ** denotes P<0.01, *** denotes P<0.001 and **** denotes P<0.0001.

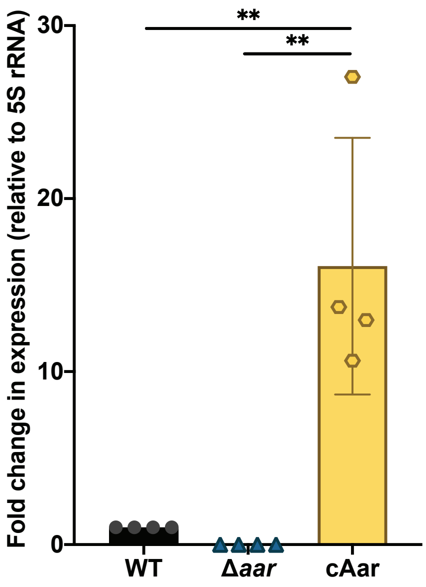

Supplementary Figure 14: Quantification of Aar expression levels in *A. baumannii* wildtype (WT; carrying pWH1266), the *aar* deletion strain (Δ*aar*; expressing pWH1266) and an *aar* complementation strain (cAar) as determined by northern blotting. Aar expression levels were first adjusted to the loading control (5S rRNA). The fold-change in Aar expression levels was then compared to the control sample (WT, set to 1). Error bars represent the standard deviation from four independent biological replicates. Statistical comparisons were performed using One-way ANOVA followed by Tukey's multiple comparisons test. Differences were considered statistically significant where ** denotes $P<0.01$.

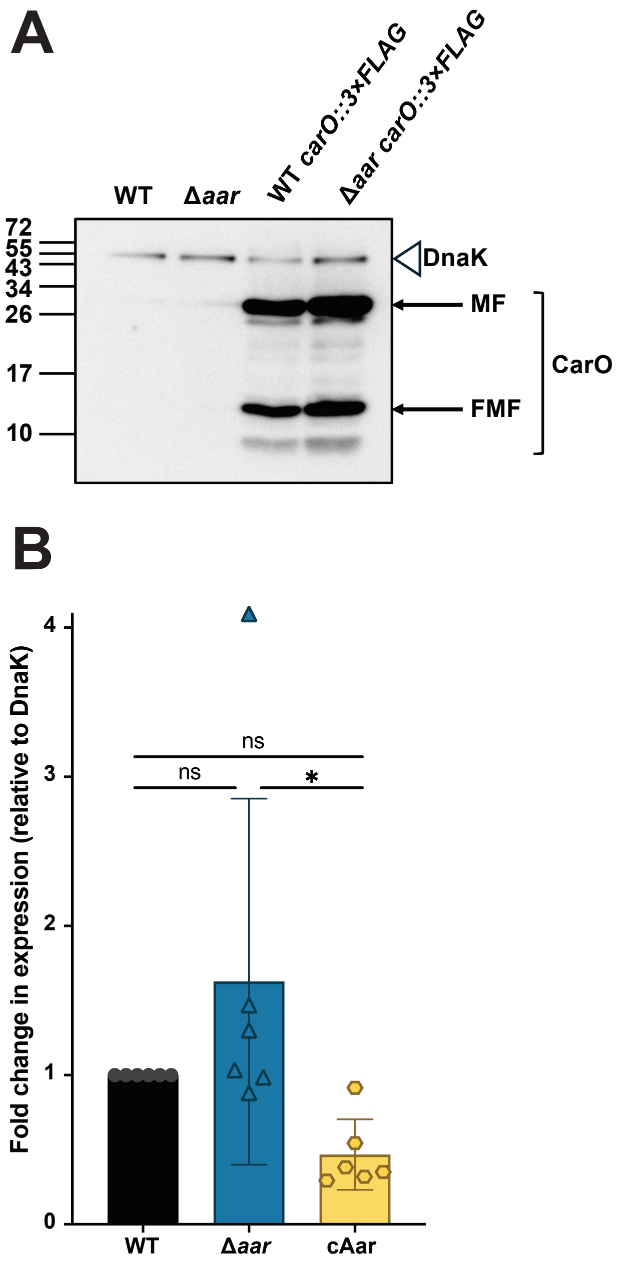

Supplementary Figure 15: Investigating regulation of CarO by Aar. (A) The expression of CarO in *A. baumannii* wildtype (WT), *aar* deletion (Δ*aar*), wildtype and *aar* deletion chromosomal C-terminal CarO::3×FLAG fusions (WT *carO*::3×FLAG and Δ*aar carO*::3×FLAG, respectively) as determined by western blotting. Whole cell lysates were isolated from *A. baumannii* grown to LSP. (B) Quantification of chromosomal CarO protein levels in *A. baumannii* wildtype (WT; carrying pWH1266), the *aar* deletion strain (Δ*aar*; carrying pWH1266) and an *aar* complementation strain (cAar) as determined by western blotting. CarO levels were first adjusted to the DnaK loading control. The fold change in CarO protein levels was then compared to the control sample (WT, set to 1). Error bars represent the standard deviation from six independent biological replicates (n=6). Statistical comparisons were performed using One-way ANOVA followed by Tukey's multiple comparisons test. Differences were considered statistically significant where ns denotes P>0.05 and * denotes P<0.05.
